# Selenophene-Modified Boron Dipyrromethene-Based Photosensitizers Exhibit Photodynamic Inhibition on a Broad Range of Bacteria

**DOI:** 10.1021/acsomega.2c02868

**Published:** 2022-09-16

**Authors:** Ahmet Caglar Ozketen, Osman Karaman, Alara Ozdemir, Isil Soysal, Caglayan Kizilenis, Aisegkioul Nteli Chatzioglou, Yagiz Anil Cicek, Safacan Kolemen, Gorkem Gunbas

**Affiliations:** † Department of Chemistry, Middle East Technical University, Ankara 06800, Turkey; ‡ Department of Chemistry, Koc University, Istanbul 34450, Turkey; § Biochemistry Graduate Program, Middle East Technical University, Ankara 06800, Turkey

## Abstract

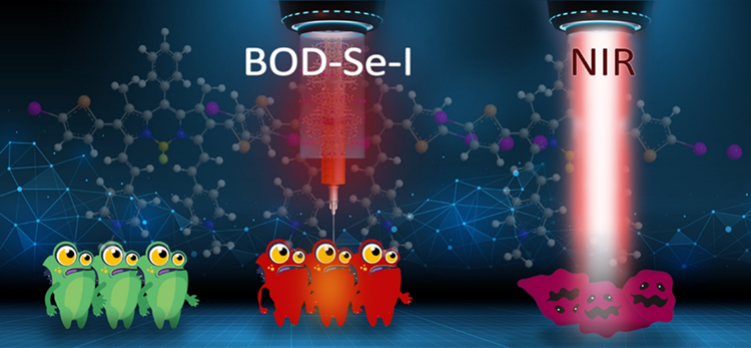

Microorganisms are crucial for human survival in view of both mutualistic and pathogen interactions. The control of the balance could be achieved by use of the antibiotics. There is a continuous arms race that exists between the pathogen and the antibiotics. The emergence of multidrug-resistant (MDR) bacteria threatens health even for insignificant injuries. However, the discovery of new antibiotics is not a fast process, and the healthcare system will suffer if the evolution of MDR lingers in its current frequency. The cationic photosensitizers (PSs) provide a unique approach to develop novel, light-inducible antimicrobial drugs. Here, we examine the antimicrobial activity of innovative selenophene-modified boron dipyrromethene (BODIPY)-based PSs on a variety of Gram (+) and Gram (−) bacteria. The candidates demonstrate a level of confidence in both light-dependent and independent inhibition of bacterial growth. Among them, selenophene conjugated PS candidates (BOD-Se and BOD-Se-I) are promising agents to induce photodynamic inhibition (PDI) on all experimented bacteria: *E. coli*, *S. aureus*, *B. cereus*, and *P. aeruginosa*. Further characterizations revealed that photocleavage ability on DNA molecules could be potentially advantageous over extracellular DNA possessing biofilm-forming bacteria such as *B. cereus* and *P. aeruginosa*. Microscopy analysis with fluorescent BOD-H confirmed the colocalization on GFP expressing *E. coli*.

## Introduction

Antibiotic resistance in pathogenic bacteria is a major threat to public health.^1^ In the wake of a devastating pandemic of SARS-COV-2, secondary infections of pathogenic bacteria are more probable than ever.^2^ Moreover, these bacteria are the foremost cause of mortality in patients with healthcare-related infections.^3^ Skin infections rooting from *Staphylococcus aureus* and *Pseudomonas aeruginosa* are the major cause of death in hospitalized immunocompromised patients.^4^ Overuse, inappropriate prescription, and extensive agricultural use of antibiotics gave rise to microbial organisms that have developed various mechanisms in order to resist the antibiotics.^5^ Infections of single or multiple resistant bacteria are more lethal, and antibiotic prescription is no longer an effective solution.^6^ This problem is referred to as a “silent pandemic of drug-resistant infections” and the lack of substitute treatments for antibiotics paving the way for the next global pandemic.^7,8^ Biofilm-forming bacteria possess the advantage of biofilm which serves as a protective layer to fight hostile environments containing antimicrobial agents such as antibiotics.^9^ Thus, the eradication of bacterial infection is a challenge due to biofilm formation. Stewart and his colleagues state that several mechanisms exist to contribute to the resistance due to biofilms, resulting in decreased antibiotic activity. These mechanisms include lowering diffusion of biocides, deactivating antibacterial agents via outer layers of the biofilm, exploiting drug pump as a conventional resistance mechanism, using differentiation into the protected phenotypic state, and altering the microenvironment by the depletion of nutrients or waste accumulation.^10^ Current advances in the treatment of antibiotic-resistant bacteria are not sufficient, and alternative strategies are required to address this menace. The strategies include the employment of amphipathic cationic molecules such as antimicrobial peptides,^11^ surfactants,^12^ enzymes (DNases, proteases, etc.),^13^ or photodynamic inactivation to kill bacteria.^14^

The photoinactivation of microorganisms was described by Oscar Raab for the first time in 1900 when the toxic effect of acridine on *Paramecium* in the presence of light was recorded.^15^ Photodynamic inactivation (PDI) is a noninvasive method based on the interaction of light with photosensitizers (PS) and oxygen.^16−20^ Excitation of PS with a specific wavelength of light results in reactive oxygen species (ROS), which takes part in cytotoxic pathways that result in the destruction of the desired microorganism.^21^

The PS of choice is crucial for PDI-dependent toxicity against microbes. The photosensitizer should selectively target bacteria while not inducing undesired toxicity to the healthy mammalian cells. Cationic charge on the PS is one of the efficient strategies to obtain selective targeting ability because microbial membranes are anionic in nature.^22^ Furthermore, a PS of cationic nature that could bind the targeted microbe in a rapid manner could offer a distinguishing feature to reduce unintended phototoxicity to other tissues as in cationic peptide antibiotics.^23,24^ Cationic photosensitizers can also be potential DNA photocleavers. Thanks to DNA’s negative charge, cationic dyes can be attracted to DNA. In 1990, Rajasinghe and his colleagues showed the DNA cleavage activity of superoxide radicals during ethanol metabolism.^25^ Later on, other studies with cationic dyes showed DNA cleavage from ROS generation. Among potential PS scaffolds, BODIPY derivatives have attracted great attention in the field of PDI, thanks to their long-lasting success in photodynamic therapy (PDT) applications against cancer cells.^26−29^ One remarkable advantage of BODIPY dyes is that they have an affinity toward the DNA; and in the presence of light, free radicals (specifically singlet oxygen and hydroxyl radical) can cleave DNA.^30^ As a result, it is possible that selenophene-modified BODIPY derivatives also have a DNA cleavage activity.

In a recent publication, Masood et al. reported two cationic BODIPY derivatives effective against cancer cells and antibiotic-resistant *E. coli* and *S. aureus* strains.^31^ An oxygen self-supplying BODIPY compound was demonstrated to inhibit *P. aeruginosa* by relieving hypoxia to boost the PDT effect.^32^ A neutral BODIPY derivative was demonstrated to kill *S. aureus*, *P. aeruginosa*, and *C. albicans* among 15 BODIPY compounds.^33^ In our previous work, we reported the mitochondria targeting BODIPY-based PDT drugs inducible in the near-infrared (NIR) range and they efficiently kill cancer cells even under hypoxic conditions.^34^ Upon excitation with far-red and near-infrared light, the selenophenyl BODIPY promotes the spin-orbit coupling interaction; therefore intersystem crossing from singlet to triplet excited state promoted, and the activity of singlet oxygen generation advanced.^35^ Among the synthesized selenophene-substituted BODIPY (**BOD-Se**, **BOD-Se-I**) PSs, **BOD-Se-I** exhibit a significant phototoxicity, which proved with the singlet oxygen quantum yield in aqueous solutions, BOD-Se-I (Φ_Δ_ = 0.32), BOD-Se (Φ_Δ_ = 0.17), and BOD-Br (Φ_Δ_ = 10%) in our previous study. **BOD-Se-I** showed a higher singlet oxygen quantum yield than the previously reported halogenated derivative of the same BODIPY core without selenophenes.^34^ In addition, BOD-Se-I and BOD-Se were nonfluorescent while BOD-Br and BOD-H were fluorescent with the yield of ϕ_F_ = 8% and ϕ_F_ = 10% in phosphate buffer (PBS) buffer (pH 7.4, 1% DMSO), respectively.^34^ The mitochondria is an important organelle responsible for oxidative phosphorylation and MT-induced apoptosis, hence an appropriate target for PDT against cancer.^36^ Moreover, the negative membrane potential of the mitochondria aids in targeting using cationic drugs.^37^ The endosymbiotic theory states that the evolutionary origin of mitochondria and chloroplasts is derived from bacterial ancestors.^38,39^ It is safe to assume that mitochondria targeting drugs could work on bacteria because of structural resemblance. Here, we demonstrate the PDI activity of BODIPY-based agents on a wide range of planktonic bacteria from both Gram (−) and Gram (+) spectrum including notorious, drug-resistant *S. aureus* and biofilm-forming *P. aeruginosa*. Microscopy images exhibit the accumulation of drugs on the membrane of Green fluorescent protein (GFP) expressing *E. coli*. We also characterized the photocleavage ability of the candidate drugs on DNA to understand the possibility of applications targeting extracellular DNA of biofilm-forming bacteria such as *Bacillus cereus* and *Pseudomonas aeruginosa*.

## Results and Discussion

### Selenophene-Modified BODIPY Derivatives Inhibit Bacterial Colony Formation

**Scheme 1:**
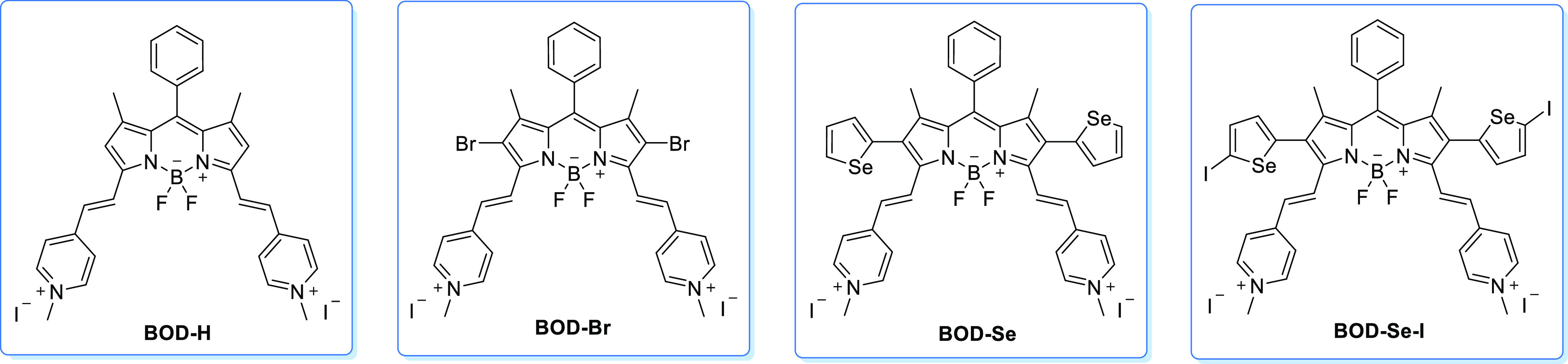
Structures of **BOD-H**, **BOD-Br**, **BOD-Se**, and **BOD-Se-I**

BODIPY is a valuable and promising skeleton to build functionalized photosensitizers. To do that, its fluorescent properties are needed to be diminished, and its triplet excited-state lifetime is needed to be boosted. The introduction of the heavy atom effect to the system is an efficient strategy to achieve this enhancement.^40−42^ Introducing the selenium atom as a heavy atom resulted in higher singlet oxygen generation and enhanced cytotoxicity toward tumor cells compared to its Bromo analogue as a result of enhanced heavy atom mediated spin-orbit coupling induced inters-system crossing (ISC). Furthermore, an extension of the absorption maximum was achieved by introducing the selenophene ring.^34^ In light of valuable data obtained in our previous work, it was envisioned that these photosensitizers could have photodynamic inhibition on bacteria, and results revealed that target photosensitizers have a decent PDI effect on a wide range of bacteria. The photodynamic inhibition (PDI) of BODIPY derivatives was assayed on two different types of Gram (−) and Gram (+) bacteria specifically; *E. coli*, *P. aeruginosa*, *B. cereus*, and *S. aureus*, respectively. The bacteria were treated with certain concentrations (0.001, 0.005, 0.01, 0.05, 0.5, and 5 μM) of **BOD-Br**, **BOD-Se-I**, and **BOD-Se** (Scheme 1). The concentrations were determined by taking advantage of the previous report stating the maximum concentration of unintended toxicity on eukaryotic cell lines.^34^ All of the three candidates showed effective photodynamic antibacterial inhibition in different bacteria and different concentrations.

The iodine-conjugated **BOD-Se-I** is a promising candidate and effective on all bacterial strains we here studied (Table 1). At least 99.9% or 3 log10 inhibition should be achieved for a candidate agent to be considered as antimicrobial or bactericidal.^43^ It was shown that **BOD-Se-I** can reduce microbial growth with 4 log10 ratios for all four types of bacteria. The **BOD-Se-I** is efficient against both *S. aureus* and *B. cereus* at 10 times lower concentration (50 nM) compared to the minimum concentration detected in dark toxicity. The result indicates photodynamic inhibition provides light-inducible enhanced activity on Gram (+) bacteria even at low concentrations. **BOD-Se-I** has distinct antimicrobial activity on Gram (−) *E. coli* compared to other drug candidates upon light treatment. More to the point, the biofilm-forming *P. aeruginosa* is susceptible to its bactericidal activity induced by light. The second efficient candidate is **BOD-Se**, which has inhibitory activity on all four strains of bacteria without the requirement of light. However, it exhibits much stronger bactericidal efficacy combined with light. **BOD-Se** showed a noteworthy bactericidal effect at low concentrations in Gram (+) compared to Gram (−) strains. However, the light-independent toxicity for **BOD-Se** is also higher than that for **BOD-Se-I** for Gram (−) *E. coli* and *P. aeruginosa*. Nonselenophene bromine-conjugated **BOD-Br**, likewise, exhibits significant antimicrobial photodynamic inactivation (aPDI) activity in Gram (+) strains with significant dark toxicity in higher concentrations. In Gram (−) strains, it effectively killed *P. aeruginosa* in higher concentrations (5000 nM); however, it failed to kill *E. coli*. Therefore, selenophene-mediated BODIPYs are observed to inhibit bacterial growth in a more effective manner than bromine-conjugated ones. This result is in parallel with the singlet oxygen quantum yield of the selenophene-mediated BODIPY drugs.^34^ Selenium is widely used as an antimicrobial and antioxidant against a variety of pathogens.^44−48^ The involvement of selenium or heterocyclic selenophene contributes to the heavy atom effect and demonstrates enhanced singlet oxygen yield as a result of enhanced spin-orbit coupling-mediated ISC.^35,49^ Our previous work reports that selenophene addition to BOD-Se and BOD-Se-I resulted in a 7 and 22% increase in the singlet oxygen yield compared to BOD-Br, respectively. Hence, the role of Se element in the photoinactivation of the bacteria we here studied is severe.

**Table 1:** Antimicrobial Activity of BOD Derivatives against Gram (−) and Gram (+) Bacteria^a^

	*E. coli* (top 10)		*S. aureus* (ATCC 25923)		*B. cereus* (NRRL B-3711)		*P. aeruginosa* (ATCC 27853)	
	light	dark	light	dark	light	dark	light	dark
BOD-Br	ND	ND	<99.99%	<99.99%	<99.99%	<99.99%	<99.99%	ND
			4 log10 (0.05 μM)	4 log10 (5 μM)	4 log10 (0.05 μM)	4 log10 (5 μM)	4 log10 (5 μM)	
BODSe-I	<99.99%	ND	<99.99%	<99.99%	<99.99%	<99.99%	<99.99%	ND
	4 log10 (0.5 μM)		4 log10 (0.05 μM)	4 log10 (0.5 μM)	4 log10 (0.05 μM)	4 log10 (0.5 μM)	4 log10 (0.5 μM)	
BOD-Se	<99.99%	<90%	<99.99%	<99.99%	<99.99%	<99%	<99.99%	<99%
	4 log10 (5 μM)	1 log10 (5 μM)	4 log10 (0.05 μM)	4 log10 (5 μM)	4 log10 (0.05 μM)	2 log10 (0.5 μM)	4 log10 (0.5 μM)	2 log10 (5 μM)

a The percentage of killed bacteria on plates is presented. The concentration is given between bracelets. ND, not detected a significant number of CFU changes.

### MTT Assays Support the Antimicrobial Activity of PDT Candidates

**Figure 1:**
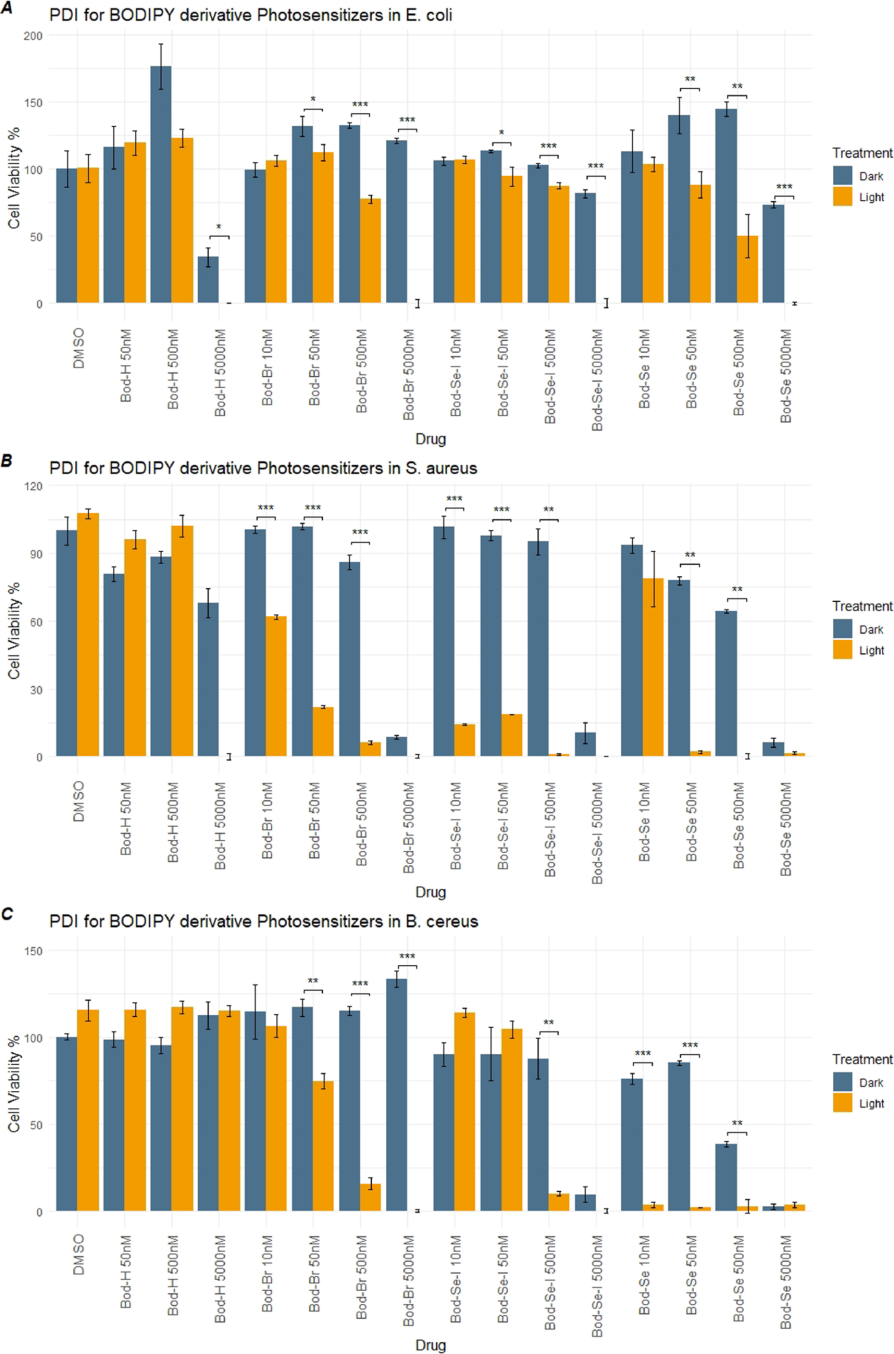
MTT assays show the percentage of the cell viability of the bacteria (A: *E. coli*, B: *S. aureus*, C: *B. cereus*) after the treatment. Dark blue: Light-independent, yellow-orange color: light-dependent cytotoxicity on the indicated bacteria. (Student’s *t*-test; *p-value ≤0.05, **p-value ≤0.01, ***p-value ≤0.001).

MTT (3-(4,5-dimethylthiazol)-2,5-diphenyltetrazolium bromide) tests are conducted to measure the antimicrobial activity of PDT candidates to further support the results of PDI ability. The bacteria are assayed on 96-well plates to measure the formazan formation due to metabolic activity. Figure 1 represents the graphical summary of the MTT results which are in parallel with the results in Table 1. Although the starting CFU number is 100 times higher than the assay for inhibition of colony formation; the results standstill and reveal the significant inhibition of cell viability upon PDI. The superior activity of PDI drug candidates of selenophene-substituted BODIPYs compared to the BOD-Br and BOD-H (heavy atom-free analogue, Scheme 1) is recorded with the MTT tests. The result showed that the 500 nM BOD-Se-I has higher phototoxicity than BOD-Se on Gram (+) bacteria. In addition, BOD-Se-I has a better inhibitory effect on Gram (+) *S. aureus*. The results also reveal that BOD-Se is more efficient in Gram (+) *B. cereus* in terms of minimum concentration of inhibition. Hence, BOD-Se-I and BOD-Se exhibit stronger aPDI activity consistent with the result of their singlet oxygen production and the results shown in Table 1. However, although **BOD-Se-I** exhibited more encouraging results compared to **BOD-Se** in plate assay, MTT assays showed better performance for **BOD-Se** for different bacteria at different concentrations. Although MTT assay is valuable to screen antimicrobial activity in a high-throughput manner, the results may differ depending on the initial cell number and the type of the bacterial species depending on several factors.^50^ For PDI action specifically, Park et al. also reported that photosensitizers (porphyrin-based in this study) can induce rapid decolorization of MTT formazan under light.^51^ The underlying mechanism for our specific case is not clear to us at the moment; however, studies along this line are currently underway. It is clear, however, that the selenophene containing BODIPY dyes are quite effective for PDI in different bacteria. We could not present a reproducible graph for *P. aeruginosa* because of the slimy nature of biofilm formation interfering with the spectrophotometric reading of formazan crystals. The results indicate the efficacy of the usage of PDT candidates for their apparent PDI activities.

The observed outcome suggests the presence of different drug response for different bacteria species. Each species of bacteria has distinct physiological and biochemical structures along with the common features such as peptidoglycan enriched cell walls in Gram-positive or lipopolysaccharide (LPS) and outer membrane presence in Gram-negative bacteria. These common and distinct features affect the mode of action of a drug and the range/spectrum of susceptible bacteria. We have observed all compounds successfully inhibit Gram-positive bacteria and *P. aeruginosa*, whereas selenophene-modified compounds (BOD-Se-I and BOD-Se) are more efficient to kill *E. coli* than BOD-Br. The porous nature of Gram-positive cell walls may result in susceptibility for this observation parallel with the literature.^52,53^ However, the success of BOD-Se and BOD-Se-I can be attributed to high singlet oxygen yield or cationic properties of methyl pyridinium groups against Gram-negative *E. coli*, as discussed in the study of Liu et al.^53^

In the Supplementary Table 1, we have listed some of the previously published BODIPY dyes to compare the efficacy. Although some of the BODIPY derivatives listed in Supplementary Table 1 shown to have PDI effect at lower concentration compared to our BODIPY derivatives, BOD-Se-I and BOD-Se are effective against four different bacteria species demonstrating a broader range of bacteria spectrum compared to the literature examples.

### Confocal Microscopy Analysis Uncovers the Localization of BOD-H on GFP Expressing *E. coli*

**Figure 2:**
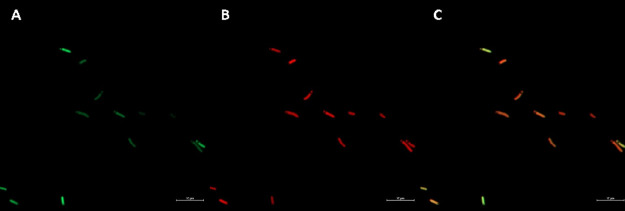
Subcellular localization of BOD-H in GFP expressing *E. coli* K12. (A) eGFP, (B) BOD-H, and (C) Merged images are recorded after 10 min dark incubation followed by washing with PBS buffer. Fluorescence excitation and emission wavelengths were at 596 nm for eGFP and 613 nm for TexasRed (Zeiss LSM900 with airyscan, magnification at 63×).

The imaging of bacteria contributes to the study of subcellular localization patterns and mode of action analysis. To demonstrate the affinity of fluorescent **BOD-H** of the PSs studied in this work, to bacteria, we employ the genetically modified *E. coli* K12 strain that has green fluorescent protein-expressing plasmid (pSB1C3-GFP). The results show a clear merged yellow-orange color formation in the overlay image in Figure 2. The localization pattern signifies the colocalization of the bacteria with **BOD-H**. The results are consistent with observed mitochondrial localization in the studies conducted on cancer cells.^34^ The bacteria-like membrane structure of the mitochondria and the **BOD-H** have an affinity to each other as well as bacteria itself. The result demonstrates the imaging ability of **BOD-H**, which could be exploitable as a staining dye in colocalization studies on bacteria. The result also suggests the mechanism of inhibitory activity may arise from the oxidation of cell membrane lipids. The imaging of the candidates **BOD-Br**, **BOD**-**Se**, or **BOD-Se-I** is not possible because of their enhanced ROS production, resulting in a decreased amount of fluorescence yield as these two are competing pathways.

### Candidate PDT Agents Exhibit ROS-Dependent Inhibitory Activity

The ability to release ROS (exclusively singlet oxygen) is emphasized in the previous work of anticancer properties.^34^ The inhibitory mechanism may be related to direct oxidation of nearby structural components such as lipid peroxidation as well as producing ROS to the environment. We conducted an experiment using known ROS inhibitors such as NaN_3_ (10 mM) for singlet oxygen quencher and KI (10 mM) for OH radicals.^22,30,54^ The observation of a decrease in PDI activity on bacteria is commented as dependency on ROS molecules. The enhanced heavy atom effect of **BOD-Se-I** indicates a better ^1^O_2_ generation yield compared to other drug candidates on both normoxic and hypoxic conditions in cell culture studies as we mentioned in our previous study.^34^ Thus, it will make our candidate suitable to test as a ROS-dependent inhibitory PS on bacteria. Hence, we assayed the activity of **BOD-Se-I** in the same conditions as before, except for the presence of ROS inhibitors and PBS as a negative control. Tables 2 and 3 represent the PDT activity of **BOD-Se-I** in *E. coli* and *S. aureus*. Significantly, **BOD-Se-I** (0.5 μM) is strongly dependent on singlet oxygen and OH radicals in *E. coli*. The inhibitory activity significantly halted after the addition of ROS inhibitors to the environment. However, the results observed for *S. aureus* indicate that the ROS inhibition is not sufficient to halt bacterial growth. Possibly, the inhibition resulted from the material’s own toxicity that was able to prevent microbial growth. Thus, the inhibition effect of **BOD-Se-I** on *S. aureus* was not observed when ROS species are scavenged by KI or NaN_3_, as listed in Table 3.

**Table 2:** ROS Inhibitors Assay on Gram (−) *E. coli*^a^

	DMSO		BOD-Se-I	
	light	dark	light	dark
NaN_3_ (10 mM)	ND	ND	ND	ND
KI (10 mM)	ND	ND	ND	ND
PBS	ND	ND	<99.99% (0.5 μM)	ND

a NaN_3_ is an inhibitor of singlet oxygen, whereas KI is an inhibitor of OH radicals. The percentage of killed bacteria on plates is presented. The concentration is given between bracelets. Only **BOD-Se-I** is studied because of its unique antimicrobial activity. ND, not detected any significant CFU change; PBS, phosphate buffer.

**Table 3:** ROS Inhibitors Assay on Gram (+) *S. aureus*^a^

	BOD-Br		BOD-Se-I		BOD-Se		DMSO	
	light	dark	light	dark	light	dark	light	dark
NaN_3_ (100 mM)	ND	ND	<99.99% (0.05 μM)	ND	<99.99% (0.05 μM)	ND	ND	ND
KI (100 mM)	<99.99% (0.05 μM)	ND	<99.99% (0.05 μM)	ND	<99.99% (0.05 μM)	ND	ND	ND
PBS	<99.99% (0.05 μM)	ND	<99.99% (0.05 μM)	ND	<99.99% (0.05 μM)	ND	ND	ND

a NaN_3_ is the inhibitor of singlet oxygen, whereas KI is the inhibitor of OH radicals. The percentage of killed bacteria on plates is presented. The concentration is given between bracelets. ND, not detected any significant CFU change; PBS, phosphate buffer.

### PDT Candidates Reveal Photocleavage Activity on Plasmid DNA

**Figure 3:**
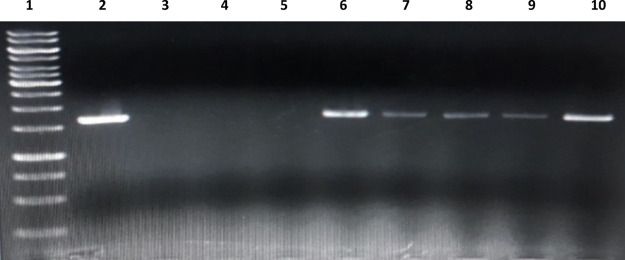
Agarose gel electrophoresis reveals photocleavage of the plasmid DNA by BOD-Br (50 μM), BOD-Se-I (50 μM), and BOD-Se (50 μM). Lane 1: Ladder; lane 2: DNA control; lane 3: DNA + BOD-Br (light); lane 4: DNA + BOD-Se-I (light); lane 5: DNA + BOD-Se (light); lane 6: DNA + DMSO (light); lane 7: DNA + BOD-Br (dark); lane 8: DNA + BOD-Se-I (dark); lane 9: DNA + BOD-Se (dark); lane 10: DNA + DMSO (dark).

One of the reasons for the notorious success of biofilm-forming bacteria is their ability to use extracellular interface as a primary defense site. Moreover, the presence of extracellular DNA provides swift action in the interaction site. Hence, some of the DNases are efficient in treating such microbiome as in cystic fibrosis.^55^ The combination of DNAse-like activity may strongly increase the activity of an antimicrobial compound.^56^ Because the PDT agents used in the study are cationic, it is tempting to say that they may show affinity to DNA. To test the hypothesis, we used plasmid DNA for light-dependent and independent activity of the candidates. As expected, the results confirm the activity of PDT reagents on the DNA when the light is applied (Figure 3). We observe negligible light-independent activity when the brightness of DNA bands on lanes 7-8-9 are compared to the negative control on lane 10. However, the clear absence of DNA in light-dependent application implicit the destruction of DNA with PDT candidates. There are several macromolecules or organelles in the cell that could be a potential target destination for inducing cell death with ROS activity. Hence, DNA is a fitting target for antimicrobial drugs as well as anticancer drugs to induce efficient inhibition. In this direction, the PDT or PDI drugs should be checked for their interaction against DNA molecules. Wang and his collaborators reported the activity of BODIPY candidates as successful DNA photocleavers by ROS generation ability.^30^ Similarly, we here observed DNA photocleavage upon treatment with selenophene-modified BODIPY candidates.

### Photocleavage Activity of PDT Candidates are Singlet Oxygen and OH Radical-Dependent

**Figure 4:**
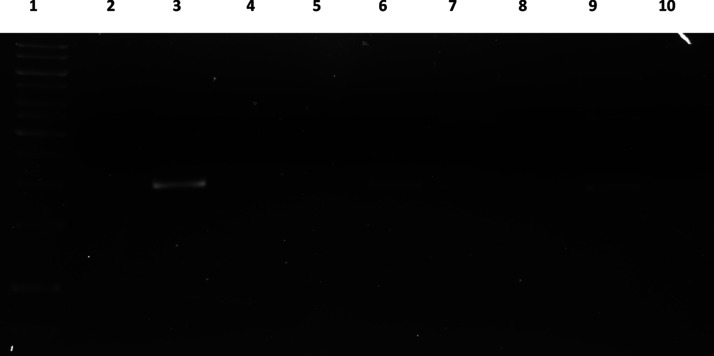
Agarose gel electrophoresis shows the inhibition of photocleavage of the plasmid DNA by BOD-Br (50 μM), BOD-Se-I (50 μM), and BOD-Se (50 μM), with KI (2 μM) and NaN3 (2 μM) ROS inhibitors. Lane 1: Ladder; lane 2: BOD-Br, lane 3: BOD-BR + NaN3, lane 4: BOD-Br + KI, lane 5: BOD-Se-I, lane 6: BOD-Se-I + NaN3, lane 7: BOD-Se-I + KI, lane 8: BOD-Se, lane 9: BOD-Se + NaN3, and lane 10: BOD-Se + KI.

To understand the mechanism of photocleavage activity on DNA, we conducted ROS inhibitors assay using NaN_3_ and KI. The results give us a hint about the mechanism of the action as the observation of DNA restored. All candidates are effective against plasmid DNA using singlet oxygen as their primary source, whereas visualization of slight band in the presence of KI indicates OH radicals are involved too (Figure 4). These results are in correlation with the previous report, suggesting strong singlet oxygen production upon light induction on PDT reagents.^34^

## Conclusions

The usage of PSs as aPDI is invaluable in the era of drug-resistant superbugs. An advantageous and efficient PS should bear a high level of phototoxicity against bacteria, whereas unintended toxicity to the healthy cell should be limited. For that purpose, we studied previously validated cationic PSs for their activity on cancer cells without harming healthy cells.^34^ The experiments conducted here clearly reveal the aPDI activity of selenophene-modified BODIPY-based PSs(BOD-Se and BOD-Se-I) have higher effect on four different bacteria strains belonging to both Gram-negative and positive categories in certain concentrations. The study also gives clues about the ROS-dependent mechanism of the mode of action against the tested bacteria. Furthermore, the possible application area for **BOD-H** as a bacterial imaging reagent is demonstrated using confocal microscopy for future work. We have demonstrated the PS activity of ROS-mediated photocleavage on plasmid DNA. The study serves as an example of mitochondria targeting PDT drug candidates that may also be useful to eradicate microbial threats even on superbugs.

## Material and Methods

### Bacterial Strains and Growth Conditions

Two Gram (−) bacteria and two Gram-positive bacteria were selected to assay PDI. *E. coli* Top10 strain is purchased from Invitrogen Co., *P. aeruginosa* ATCC 27853, *S. aureus* ATCC 25923, and *B. cereus* NRRL B-3711 were obtained from the American Type Culture Collection (ATCC, Rockville, MD, USA), Northern Regional Research Laboratory (NRRL, USDA, Peoria, Illinois/USA). The GFP expressing *E. coli* K12 (pSB1C3 backbone) was received as a courtesy of Sinem Ulusan. All strains of bacteria were cultured in LB medium or LB agar plates at 37 °C for overnight (16–18 h) incubation. All bacterial strains were stored with glycerol stocks at −80 °C.

### Microscopy Analysis

The protocol for imaging was conducted as described in Rice et al. with slight changes.^57^ Fresh culture of GFP expressing *E. coli* K12 was started using an inoculum from −80 °C stock and incubated at 37 °C for overnight at 200 rpm. The next day, bacteria were harvested by centrifugation at 3000 rpm for 2 min. The pellet was resuspended in 1 mL of filter-sterilized PBS buffer (pH 7.4) and centrifuged. The washing step was repeated three more times. Finally, the concentration of bacteria is adjusted to 0.7 (*A*_600nm_) with PBS buffer. BOD-H and BOD-H-Me (Final concentration: 5 μM) were added to 2 mL of bacteria each and incubated in the dark for 10 min. The excess dye was removed by washing twice in PBS buffer. Approximately, 20 μL of sample was used for confocal microscopy analysis. The samples were excited at around 596 nm and detected at around 613 nm using a Texas Red filter (with 63× magnification) on a Zeiss AIRYSCAN LSM900 confocal microscope.

### Antimicrobial Activity Assay

The method of antimicrobial activity was assayed as described in Rice et al. with slight changes.^57^ Fresh bacterial culture was started with an inoculum in sterile LB medium and incubated for overnight at 37 °C at 200 rpm. The bacteria were harvested at 3000 rpm (except for *P. aeruginosa*, centrifugation at 6000 rpm was applied because of its slimy texture) and washed four times using sterile PBS buffer. The concentration of bacteria was set to 10^8^ CFU/mL (for *E. coli*; OD_600_: 0.125, *S. aureus*; OD_600_: 0.08, *B. cereus* and *P. aeruginosa*; OD_600_: 0.5). The final concentrations (0.001, 0.005, 0.01, 5, 0.5, and 0.05 μM) of candidate drugs were mixed with 10^6^ CFU/mL in PBS buffer. The same volume of DMSO (Hybri-Max, ≥99.7%, Sigma-Aldrich CAS No: 67-68-5) was used as a negative control because the drugs dissolved in this solvent. The samples were kept in the dark for 20 min. Then, the samples (200 μL each) were transferred into a sterile 96-well plate. Light was applied exactly 10 cm of distance away from the LED source (630 nm) or kept in the dark for 1 h at room temperature. Fifty microliters (approximately 5 × 10^4^ CFU) of each well were transferred and plated on LB agar plates and incubated for overnight at 37 °C. The colonies were observed and recorded the next day.

### MTT Assay

MTT assay was conducted to assess the effect of antimicrobial activity following a protocol reported by Alenezi et al. with minor modifications.^58^ Briefly, fresh bacterial culture was prepared as stated above. Harvested cells were washed four times with PBS buffer. The concentrations of bacteria were adjusted to 10^9^ CFU/mL. The final concentrations (5, 0.5, and 0.05 μM) of candidate drugs were mixed with 10^8^ CFU/mL in PBS buffer. The same volume of DMSO (Hybri-Max, Sigma-Aldrich) was used as the negative control because the drugs dissolved in this solvent. The samples were kept at dark for 20 min. Then, the samples (100 μL each) were transferred into a sterile 96-well plate. The light was applied exactly 10 cm of distance away from the LED source (630 nm) and kept at dark for 1 h at room temperature.

Fifty milligrams of 3-(4,5-dimethylthiazol-2-yl)-2,5-diphenyl-tetrazolium bromide (MTT, 98%, Sigma-Aldrich) was dissolved in 10 mL of PBS buffer. Ten microliters of the MTT reagent and 30 μL of LB medium were added to each well and incubated for 1 h at 37 °C at 80 rpm until dark purplish color was observed. Then, 100 μL of 10% SDS solution (dissolved in 10 mM HCl) was poured to solubilize the formed formazan crystals.

The samples were incubated at 37 °C at 80 rpm for overnight. Next day, the absorbance was recorded with ThermoScientific MultiScan microplate reader (ThermoScientific Co.) at 490 and 570 nm. The samples from negative controls and high concentration drugs were plated on LB agar to ensure the cell death. All experiments were conducted with at least triplicate for each independent experiment.

### ROS Inhibitors Assay

The protocol for antimicrobial assay described above was applied except for singlet oxygen trapper (NaN_3_, ≥99%, BDH Chemicals, Cat. No:30111) and OH radical trapper (KI, 99%, Sigma-Aldrich, CAS No: 7681-11-0) with a final concentration of 10 μM for *E. coli* and 100 μM for *S. aureus*. After candidate drugs and inhibitors were mixed with the bacteria of interest, 20 min of incubation at dark was performed. Then, 1 h of incubation with light (or dark) was conducted using a LED light source (630 nm) from a vertical distance of 10 cm. The results were measured either by counting of colonies formed on LB agar plates or by MTT assay. All experiments were conducted with at least triplicate for each independent experiment.

### Photocleavage Assay

pENTRY-PstCTE1 was used as the plasmid DNA material with a size of approximately 2.5 kb.^59^ The plasmid isolation from the *E. coli* Top10/pENTRY-PstCTE1 was achieved using the AnalyticJena plasmid isolation kit (innuPREP Plasmid Mini Kit 2.0) by following the manufacturer’s protocol. The drug candidates (10, 20, and 50 μM) were mixed with 40 μg of plasmid DNA in sterile PBS buffer. The mixture was transferred to the 96-well plate. NIR (630 nm) light (or dark) was exposed from 10 cm of distance for 1 h at room temperature. Then, the mixture was separated by performing agarose gel electrophoresis (1%, 60–70 V, 50–60 min) and visualized under UV light (Kodak, Gel Imaging System).

Inhibition of the photocleavage activity was tested by the addition of NaN_3_ for singlet oxygen trapping and KI for OH radical trapping as previously reported by Wang et al.^30^ The drug candidates (final concentration, 50 μM), plasmid DNA (40 μg), and inhibitors alone or mixed (2 or 10 μM) were merged in PBS buffer. NIR (630 nm) light (or dark) was applied from 10 cm of distance for 1 h at room temperature. The results were recorded by agarose gel electrophoresis.

### Statistical Analysis

All independent experiments were performed in at least three replicates. Bacterial viability of light-induced antimicrobial activity assays were compared with their respective dark control statistically. The results were represented as means of triplicate ± standard deviation (SD) calculated from the data set of each independent experiment. Welch’s two-sample *t*-test was employed using R program to compute and indicate the statistical significance.

## Supporting Information Available

Synthetic pathway of PDT agents, comparison of PDI activity with other BODIPY derivatives, and antimicrobial activity assay photographs for lower concentrations (PDF)

## Abbreviations

MDR: multidrug resistant
PS: photosensitizer
PDI: photodynamic inhibition/inactivation
NIR: near-infrared radiation
ROS: reactive oxygen species
BODIPY: dipyrrometheneboron difluoride
PDT: photodynamic therapy
MT-induced: mitochondria induced
GFP: green fluorescent protein
FDA: U.S. Food and Drug Administration
ND: not detected
NaN_3_: sodium azide
KI: potassium iodide
OH: hydroxide
CFU: colony-forming unit
PBS: phosphate buffered saline
MTT: 3-(4,5-dimethylthiazol-2-yl)-2,5-diphenyltetrazolium bromide
