# Supplementary material for: Selenophene-Modified Boron Dipyrromethene-Based Photosensitizers Exhibit Photodynamic Inhibition on a Broad Range of Bacteria
Source: ACS Omega. 2022 Sep 16;7(38):33916–25. doi: 10.1021/acsomega.2c02868 (PMC9520714; doi:10.1021/acsomega.2c02868)
Supplement: Supplementary file 1 — ao2c02868_si_001.pdf [file ao2c02868_si_001.pdf]

Supplementary Information

Selenophene Modified BODIPY-Based Photosensitizers Exhibit Photodynamic Inhibition on Broad Range of Bacteria

Ahmet Caglar Ozketen[1], Osman Karaman[1], Alara Ozdemir[1], Isil Soysal[1], Caglayan Kizilenis[1], Aisegkioul Nteli Chatzioglou[1], Yagiz Anil Cicek[1,†], Safacan Kolemen[2], Gorkem Gunbas[1,3,*]

1. Department of Chemistry, Middle East Technical University, Ankara, Turkey

2. Department of Chemistry, Koc University, Istanbul, Turkey

3. Biochemistry Graduate Program, Middle East Technical University, Ankara, Turkey

† Current Address, Department of Chemistry, University of Massachusetts, Amherst, USA

* Corresponding Author

Synthesis of Selenophene Based BODIPY Derivatives

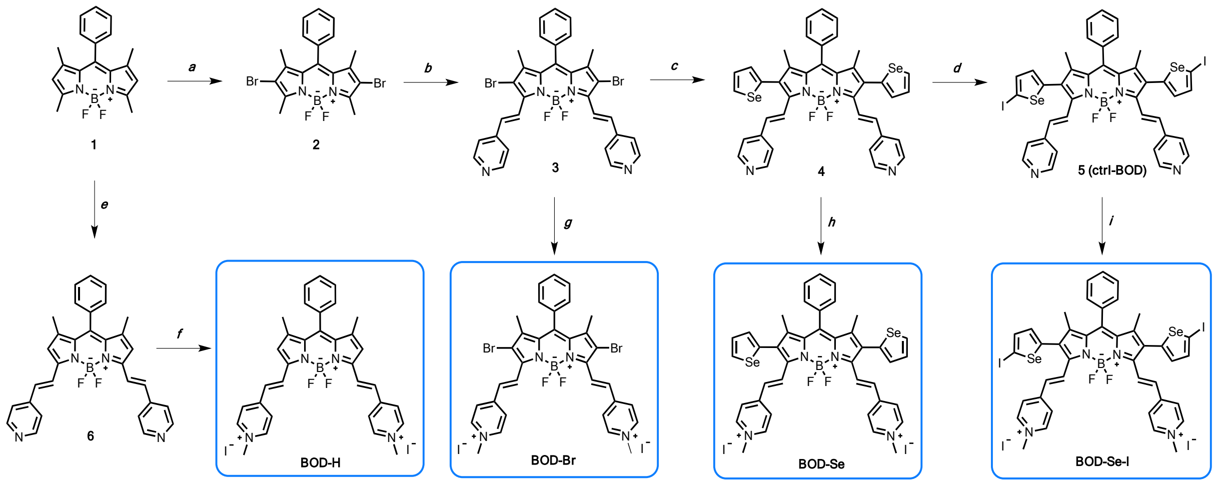

Figure S1. Detailed synthetic pathway for PDT agents. Reagents and conditions: (a) NBS, DCM, rt, 30 min, 89 %, (b) 4-pyridinecarboxyaldehyde, AcOH, piperidine, DMF, rt, 30 min, 65%, (c) tributyl(selenophen-2-yl)stannane, $Pd(PPh_3)_2Cl_2$, reflux, 5 days, 76%, (d) NIS, DCM, rt, 2 days, 83%, (e) 4-Pyridinecarboxyaldehyde, AcOH, piperidine, DMF, 40 °C, 1 day, 29%, (f) MeI, DMF, 50 °C, 2 days, 77%, (g) MeI, DMF, 50 °C, 2 days, 92%, (h) MeI, DMF, 50 °C, 1 day, 86%, (i) MeI, DMF, 50 °C, 2 hours, 87%. *ChemMedChem* **14**, 1879–1886 (2019).

Table S1: Some of the BODIPY dyes reported for PDI activity since 2018.

BODIPY derivatives	Concentration	Bacteria	Inhibition	Reference
BDP-4DPA	200 µg/mL	*S. aureus*	>1 log10	Qing et al., 2021
DP2-Br2@mPE	24 µg/mL	*E. coli*	>5 log10	Shao et al., 2020
BCNBA@ ZIF	200 µg/mL	*E. coli*	>7 log10	Lu et al., 2020
BODIPY-ZIF-8	1,25 µg/mL	*E. coli*<br>*S. aureus*	>4log10	Shen et al., 2022
BDP3-BDP5	10 nM	*S. aureus*	>4log10	Shi et al., 2022
PFH/F-I	500 µg/mL	*P. aeruginosa*	<1log10	Bai et al., 2021
B5 (Neutral BODIPY)	1 µM (*S. aureus*)<br>10 µM (*P. aeruginosa* and *C. albicans*)	*S. aureus*<br>*P. aeruginosa*<br>*C. albicans*	7log10 (*S. aureus*)<br>>4log10 (*P. aeruginosa* and *C. albicans*)	Orlandi et al., 2022
1BDPC-Py	0,1 µM (*S. aureus*)<br>0.4 µM (*E. coli*)	*E. coli*<br>*S. aureus*	>4log10	Masood et al., 2022
1BDPC-TPP	0,1 µM (*S. aureus*)<br>0.4 µM (*E. coli*)	*E. coli*<br>*S. aureus*	>4log10	Masood et al., 2022
BOD-Se-I	0,5 µM (*E. coli and P. aeruginosa*)<br>0.05 µM (*S. aureus and B. cereus*)	*E. coli*<br>*S. aureus*<br>*P. aeruginosa*<br>*B. cereus*	>4log10	Our study
BOD-Se	5 µM (*E. coli)*<br>0.5 µM (*P. aeruginosa*)<br>0.05 µM (*S. aureus and B. cereus*)	*E. coli*<br>*S. aureus*<br>*P. aeruginosa*<br>*B. cereus*	>4log10	Our study
BOD-Br	5 µM (*P. aeruginosa*)<br>0.05 µM (*S. aureus and B. cereus*)	*S. aureus*<br>*P. aeruginosa*<br>*B. cereus*	>4log10	Our study

Antimicrobial PDT activity assay for lower doses (<50nM)

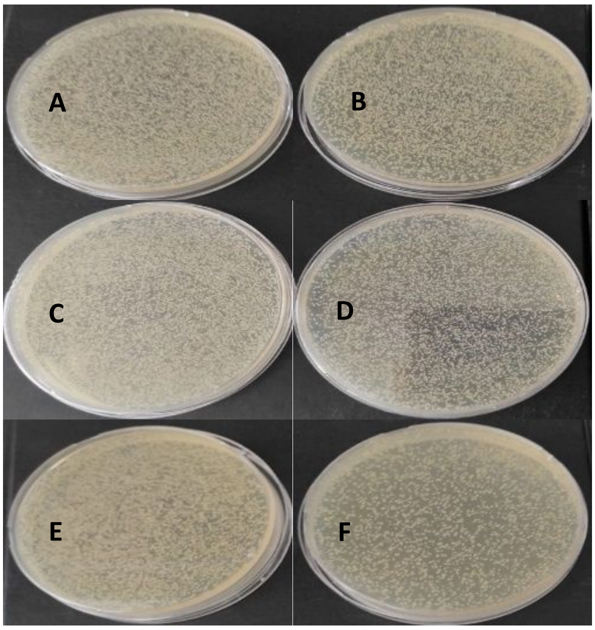

Figure S2. Antimicrobial activity assay of BOD-Se-I on *S. aureus*. A) 1nm-Dark, B) 1nm-Light, C) 5nm-Dark, D) 5nm-Light, E) 10nm-Dark, and F) 10nm-Light

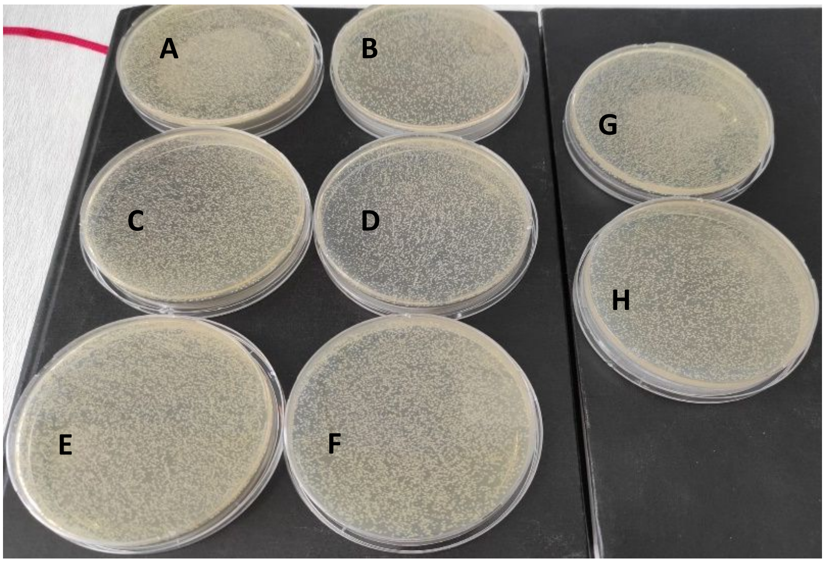

Figure S3. Antimicrobial activity assay of BOD-Br on *S. aureus*. A) 1nm-Dark, B) 1nm-Light, C) 5nm-Dark, D) 5nm-Light, E) 10nm-Dark, F) 10nm-Light, G) Control-Dark, and H) Control-Light

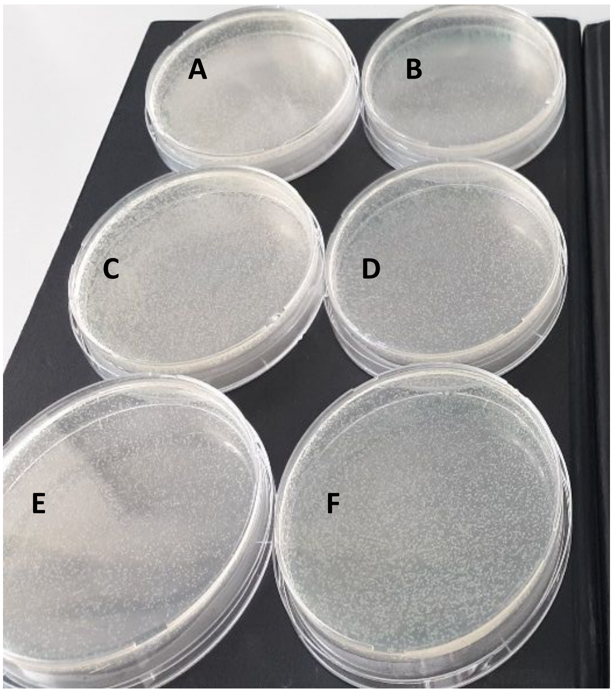

Figure S4. Antimicrobial activity assay of BOD-Se on *S. aureus*. A) 1nm-Dark, B) 1nm-Light, C) 5nm-Dark, D) 5nm-Light, E) 10nm-Dark, and F) 10nm-Light

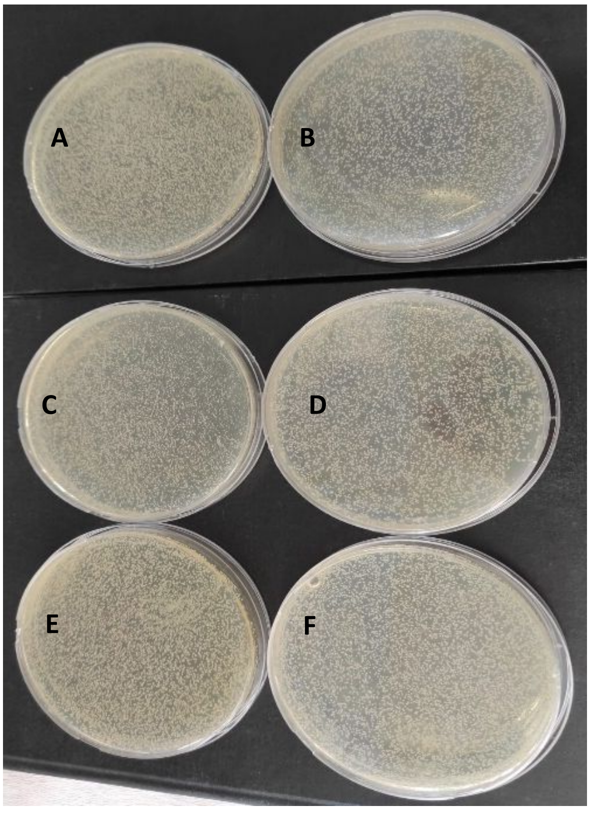

Figure S5. Antimicrobial activity assay of BOD-Se-I on *B.cereus*. A) 1nm-Dark, B) 1nm-Light, C) 5nm-Dark, D) 5nm-Light, E) 10nm-Dark, and F) 10nm-Light

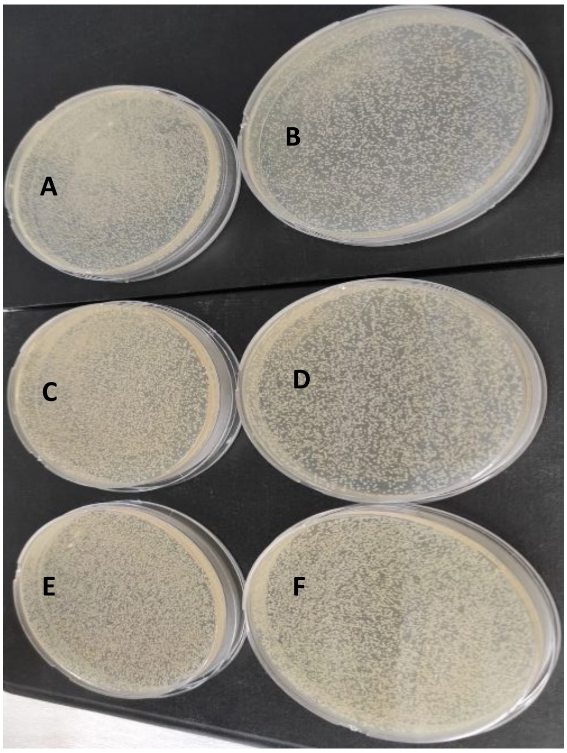

Figure S6. Antimicrobial activity assay of BOD-Br on *B.cereus*. A) 1nm-Dark, B) 1nm-Light, C) 5nm-Dark, D) 5nm-Light, E) 10nm-Dark, and F) 10nm-Light

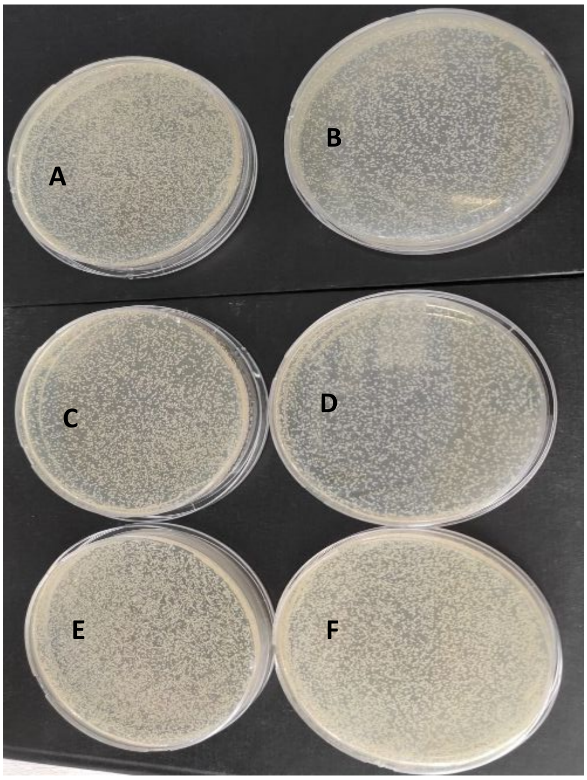

Figure S7. Antimicrobial activity assay of BOD-Se on *B.cereus*. A) 1nm-Dark, B) 1nm-Light, C) 5nm-Dark, D) 5nm-Light, E) 10nm-Dark, and F) 10nm-Light
